# Comparative Proteomic Analysis of *Rhipicephalus sanguineus* sensu lato (Acari: Ixodidae) Tropical and Temperate Lineages: Uncovering Differences During *Ehrlichia canis* Infection

**DOI:** 10.3389/fcimb.2020.611113

**Published:** 2021-01-29

**Authors:** Gustavo Seron Sanches, Margarita Villar, Joana Couto, Joana Ferrolho, Isabel G. Fernández de Mera, Marcos Rogério André, Darci Moraes Barros-Battesti, Rosangela Zacarias Machado, Gervásio Henrique Bechara, Lourdes Mateos-Hernández, José de la Fuente, Sandra Antunes, Ana Domingos

**Affiliations:** ^1^ Global Health and Tropical Medicine, Instituto de Higiene e Medicina Tropical, Universidade Nova de Lisboa (GHTM-IHMT-UNL), Lisboa, Portugal; ^2^ Escola de Ciências da Vida, Pontifícia Universidade Católica do Paraná, Curitiba, Brazil; ^3^ SaBio, Instituto de Investigación en Recursos Cinegéticos IREC-CSIC-UCLM-JCCM, Ciudad Real, Spain; ^4^ Biochemistry Section, Faculty of Science and Chemical Technologies, and Regional Centre for Biomedical Research [CRIB], University of Castilla-La Mancha, Ciudad Real, Spain; ^5^ Departamento de Patologia Veterinária, Universidade Estadual Paulista (FCAV-UNESP), Jaboticabal, Brazil; ^6^ UMR BIPAR, INRAE, ANSES, École Nationale Vétérinaire d’Alfort, Université Paris-Est, Paris, France; ^7^ Department of Veterinary Pathobiology, Center for Veterinary Health Sciences, Oklahoma State University, Stillwater, OK, United States

**Keywords:** ticks, proteomics, mialome, sialome, vector competence, *Ehrlichia*, *Rhipicephalus sanguineus*

## Abstract

The tick vector *Rhipicephalus sanguineus* is established as a complex of closely related species with high veterinary-medical significance, in which the presence of different genetic, morphological, and biological traits has resulted in the recognition of different lineages within taxa. One of the most striking differences in the “temperate” and “tropical” lineages of *R. sanguineus* (s.l.) is the vector competence to *Ehrlichia canis*, suggesting that these ticks tolerate and react differently to pathogen infection. The present study addresses the SG and MG proteome of the *R. sanguineus* tropical and temperate lineages and compares their proteomic profile during *E. canis* infection. Batches of nymphs from the two lineages were allowed to feed on naïve and experimentally *E. canis* infected dogs and after molting, adults were dissected, and salivary glands and midgut tissues separated. Samples were screened for the presence of *E. canis* before proteomic analyses. The representation of the proteins identified in infected and non-infected tissues of each lineage was compared and gene ontology used for protein classification. Results highlight important differences in those proteomic profiles that added to previous reported genetic, biological, behavioral, and morphological differences, strengthening the hypothesis of the existence of two different species. Comparing infected and non-infected tissues, the results show that, while in midgut tissues the response to *E. canis* infection is similar in the salivary glands, the two lineages show a different pattern of protein representation. Focusing on the proteins found only in the infected condition, the data suggests that the cement cone produced during tick feeding may be implicated in pathogen infection. This study adds useful information to the debate on the controversial *R. sanguineus* systematic status, to the discussion related with the different vectorial competence occurring between the two lineages and identifies potential targets for efficient tick and tick-borne disease control.

## Introduction

Ticks and tick-borne diseases are increasing concerns in human and animal health, causing a significant negative socioeconomic impact throughout the world (Jongejan and Uilenberg, 2004). The canine monocytic ehrlichiosis (CME) is a serious and potentially fatal infectious disease caused by the Gram-negative obligatory intracellular bacterium *Ehrlichia canis*, which infects monocytes and macrophages of wild carnivores and dogs (Ewing, 1969). It is probably the most widely distributed canine vector-borne disease, with high prevalence, especially in animals from tropical and subtropical areas (Ewing, 1972; Irwin, 2001; Suksawat et al., 2001).

Although the transmission of *E. canis* by the American dog tick, *Dermacentor variabilis,* has been experimentally demonstrated (Johnson et al., 1998), *Rhipicephalus sanguineus* sensu lato continues to be considered as its main biological vector to dogs (Ewing, 1969; Groves et al., 1975). For a long time, *R. sanguineus* was considered a single taxon but currently, is accepted the existence of at least two genetic lineages under the name “*Rhipicephalus sanguineus*,” one known as the temperate lineage and the other one, as the tropical lineage (Moraes-Filho et al., 2011; Nava et al., 2018). These lineages are morphologically very similar (Oliveira et al., 2005; Dantas-Torres et al., 2013), with strong differences in biology (Szabó et al., 2005; Sanches et al., 2016), and in vector competence (Cicuttin et al., 2015; Moraes-Filho et al., 2015). The most common method for controlling ticks and tick-borne diseases is the use of acaricides but, as it is widely known, these chemicals contribute strongly to environmental contamination, selection of resistant tick specimens and are often associated with high costs, highlighting the need for better control solutions (Domingos et al., 2013). Also, dogs do not develop natural protective immunity against *R. sanguineus ticks* (Évora et al., 2015) and currently, there is no commercial vaccine available to protect dogs against ticks or against the CME (Rodríguez-Mallon, 2016). The development of a vaccine targeting both tick fitness and pathogen transmission is an attractive and desired measure, which involves the identification of efficient antigens only possible with a good knowledge on host-tick-pathogen interactions (de la Fuente et al., 2016; Villar et al., 2017).

Despite being a relatively recent approach, proteomics has already greatly contributed to the field of anti-tick vaccine development enabling the identification of differentially represented proteins related to tick-pathogen interactions (Villar et al., 2017; Antunes et al., 2018; Antunes et al., 2019).

Knowing that only the tropical lineage of *R. sanguineus*, but not the temperate one, proved to be a competent vector of *E. canis* to dogs, (Moraes-Filho et al., 2015), this exploratory study was designed to identify proteins related to vector competence of the *R. sanguineus* tropical lineage. For this purpose, the sialome and the mialome of the tropical and the temperate lineage of *R. sanguineus* in non-infected and *E. canis-*infected conditions were obtained by reversed phase liquid chromatography–mass spectrometry (RP-LC-MS/MS), and further analyzed. Special interest was given to proteins present only in the infection condition, as they may be involved in processes related with vector competence and consequently, be further explored as potential targets to control *E. canis* infection in *R. sanguineus* tropical lineage.

The results obtained here represent a step forward in the knowledge of the mechanisms responsible for the difference in vector competence displayed by the two *R. sanguineus* lineages to *E. canis* transmission and identify molecules with potential to be used in the development of a vaccine targeting the tick-pathogen transmission.

## Materials and Methods

### Experimental Design and Rationale

It was previously demonstrated by Moraes-Filho et al. (2015) that the *R. sanguineus* temperate lineage lacks vectorial competence for *E. canis*. The present study hypothesizes that the vector competence of *R. sanguineus* tropical lineage correlates with molecular drivers which can be identified and further evaluated towards infection control. Thus, both the SG (infection transmission) and MG (infection acquisition) tissues were herein focused and subjected to proteomic analyses to recognize significant differences between the two lineages. For proteomics, a pool was made with all samples belonged to each group, reducing the variability between ticks and highlighting the possible differences between lineages. Underpinning these molecular drivers is key to, not only clarify the relevance of separating these groups into distinct species but also to deliver novel tick antigens involved in pathogen acquisition and/or transmission. It is important to emphasize that independent comparative proteomics analysis were conducted, focusing first the *R. sanguineus* lineages in a non-infection status and after in infected *R. sanguineus* lineages. Such approach does not allow the direct comparison of infected *vs* non-infected samples but abides the identification of proteins exclusively represented in the infect condition.

### Ethics Statement

This study was approved by the Ethics Committee on Animal Experiments (CEUA) of the School of Agricultural and Veterinary Sciences of Universidade Estadual Paulista (UNESP), Jaboticabal, SP, Brazil, process number 000800/18. Animal experiments were conducted according to the principle of the 3Rs, to replace, reduce, and refine the use of animals for scientific purposes. During the experiment, medical routine checks were daily undertaken to monitor the overall health of the animals. After the experiment, *E. canis* infected dog received doxycycline therapy (10 mg/kg, 12/12 h P.O., for 30 days). During 4 weeks after the end of the treatment, the animals were certified as free from *E*. *canis* infection (tested by qPCR).

### Ticks

*Rhipicephalus sanguineus* ticks used in this study were obtained from pathogen-free colonies maintained at the Immunopathology Laboratory of Department of Veterinary Pathology, School of Agricultural and Veterinary Sciences (FCAV) of Universidade Estadual Paulista (UNESP), Jaboticabal, SP, Brazil. The *R. sanguineus* tropical lineage colony was started from engorged females collected from healthy dogs in Jaboticabal, SP, Brazil (21°15′22′′S; 48°18′58′′W), while the *R. sanguineus* temperate lineage colony was set up from engorged females collected from dogs in Porto Alegre, RS, Brazil (30°1’40”S, 51°13’43”W). Both colonies are periodically renewed with the inclusion of local tick samples. For colony maintenance, *R. sanguineus* instars were reared on 5–8 months-old white New Zealand rabbits provided by the Central Animal Facility of UNESP, Botucatu, SP, Brazil. Tick colonies of both *R. sanguineus* lineages were separately reared in the laboratory, as previously described (Bechara et al., 1995). For their maintenance, ticks were kept in clear plastic tubes with a perforated lid in a Biochemical Oxygen Demand (B.O.D.) incubator, set for a constant temperature of 27°C, relative humidity of 90%, and 12-h light:12-h dark photoperiod.

### Hosts

Two three-month-old male German shepherd dogs (*Canis lupus familiaris)*, free of pathogens and ectoparasites, were acquired from a certified breeder, held in individual boxes at the Experimental Kennel of the Department of Veterinary Pathology, FCAV-UNESP, and fed with commercial dry food Dog Chow (Nestlé Purina, Ribeirão Preto, SP, Brazil) twice a day and water *ad libitum.* The animals were dewormed with Drontal Plus ^®^ (Bayer Animal Health, São Paulo, Brazil) and vaccinated with Vanguard Plus V10^®^ (Zoetis, New Jersey, USA) with a 3-week interval.

Before starting the experiment, 5 ml of blood were taken from each animal and tested to ensure the absence of pathogens. Part of the blood were tested by Indirect Fluorescent Antibody Test (IFAT) for specific antibodies against *E. canis* (André et al., 2010) and *Babesia vogeli* (Furuta et al., 2009), which may be as well vectored by *R. sanguineus*. Blood was also tested for the presence of *E. canis*  (Doyle et al., 2005) and *B. vogeli*  (Jefferies et al., 2007) DNA, by qPCR and PCR, respectively. Genomic DNA was extracted from the whole blood using the DNeasy Blood & Tissue Kit (Qiagen, Hilden, Germany), according to the manufacturer’s instructions.

### Infection of Dogs with *Ehrlichia canis*

Frozen infected blood (10^3^ copies of *E. canis*/µl) of third canine passage, derived from a dog previously inoculated with the Jaboticabal strain of *E. canis* (GenBank no. DQ401044) was used as inoculum to perform the experimental infection in one of the dogs. When the experimental infection took place, the dog was 6 months old. This dog was inoculated intravenously with 4 ml of defrosted infected blood heated at 37°C in a water bath. The second dog was not inoculated, being used for the feeding of ticks in non-infected condition. After inoculation, and during the whole experiment, the infection was monitored by quantitative polymerase chain reaction (qPCR) targeting a 378 bp fragment of the disulfide bond formation protein (*dsb*) gene (Doyle et al., 2005). Giemsa-stained capillary blood smears were also performed to detect the presence of morulae and observed under an Olympus CX31 light microscope (Olympus, Tokyo, Japan). For this purpose, blood samples were taken from the ear tip, until day 13 post-inoculation, when the parasitemia was confirmed.

### Experimental Groups for Proteomics Assay

At day 14 post-inoculation (one day after the parasitemia peak), 1,000 nymphs of each *R. sanguineus* lineages were placed separately in different chambers to feed on non-infected and infected dogs. Feeding chambers were daily inspected and engorged nymphs were placed in clear plastic tubes maintained in an incubator at 27°C, relative humidity of 90%, and a 12-h light: 12-h dark photoperiod, until 30 days after molt to adult. Female ticks of both lineages, obtained from nymphs fed on non-infected and infected dogs, were immersed in RNAlater (Ambion, Austin, TX, USA) and stored at −80°C, until the dissection of tick´s salivary glands (SG) and midgut (MG). Before the dissection, RNAlater was replaced to phosphate-buffered saline (PBS) pH 7.4 to avoid interference on proteomics. The dissection of tick’s SG and MG was performed in ice-cold PBS containing a protease inhibitor cocktail set II (Calbiochem, Merck, Darmstadt, Germany).

Eight experimental groups were formed: (1) Non-infected SG from the tropical lineage; (2) Non-infected SG from the temperate lineage; (3) Non-infected MG from the tropical lineage; (4) Non-infected MG from the temperate lineage; (5) Infected SG from the tropical lineage; (6) Infected SG from the temperate lineage; (7) Infected MG from the tropical lineage, and (8) Infected MG from the temperate lineage. Each group was composed by four pools containing *R. sanguineus* tick organs dissected from eight individuals.

**Figure 1:**
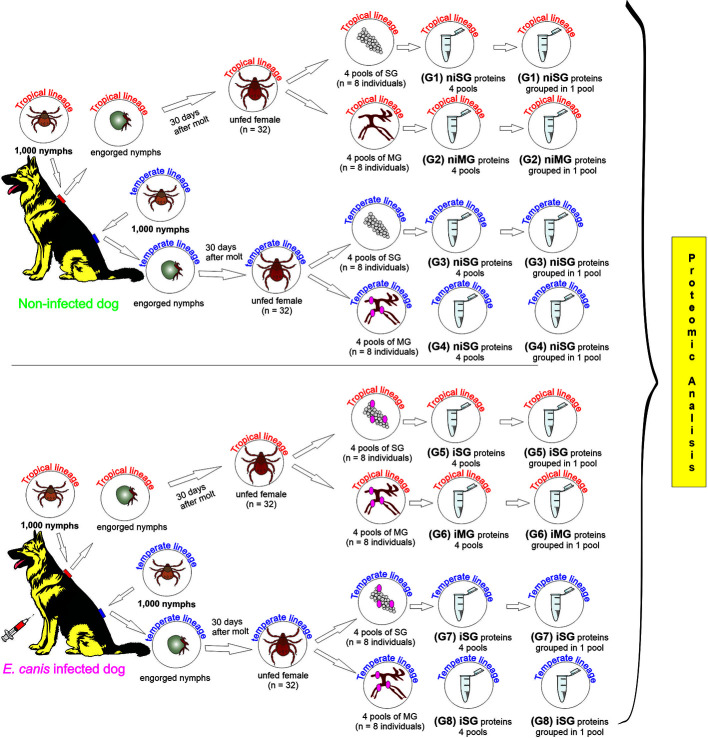
Study schema outlining the derivation of the samples, and the experimental groups subjected to the proteomic analysis. ni, non-infected condition; i, infected condition; SG, salivary glands; MG, midgut; G1–G8, experimental groups.

DNA and protein extraction from each pool of SG and MG was performed separately, and immediately after dissection, using the Qiamp AllPrep DNA/RNA/Protein extraction Mini Kit for tissue protocol (Qiagen, Hilden, Germany). The samples were first disrupted and homogenized in 350 µl RTL lysis buffer (provided in the kit), using a mortar and pestle. The subsequent steps were carried out according to the manufacturer’s instructions. The DNA concentration was estimated using ND-1000 Nanodrop Spectrophotometer (Nanodrop, Thermo Scientific, USA) and purity was checked by evaluating the absorbance ratio at 260/280. Bradford protein assay was used to measure the concentration of total protein in each pool. All samples were stored at −20°C until use. The study schema is presented in **Figure 1**.

### Non-Infection and Infection Conditions Confirmation by Polymerase Chain Reaction

To confirm the DNA integrity and the absence of PCR inhibitors, a conventional PCR targeting a 460 bp fragment of the gene encoding the small subunit ribosomal RNA (*16S rRNA*) of ticks, was performed using primers and thermal conditions previously described (Black and Piesman, 1994). The DNA amplification was carried out in a T100™ Thermal Cycler (BioRad, CA, USA), in 25-μl reaction volumes including 12.5 μl of NZYTaq II 2x Green Master Mix (Nzytech, Lisbon, Portugal) containing NZYTaq II DNA polymerase, dNTPs, MgCl_2_ and loading dye, 1 μM of each primer, 1 μl of DNA template, and nuclease-free water up to the final volume. Negative control was prepared with no template.

For the validation of the non-infection and infection condition, DNA from each pool of samples was screened for the presence of *E. *canis* *by nested PCR targeting a fragment of the *16S* rRNA gene using primers and cycling conditions based on described protocols (Murphy et al., 1998). Briefly, in 25 μl reaction volumes including 12.5 μl of NZYTaq II 2x Green Master Mix (NZYTech, Lisbon, PT) containing NZYTaq II DNA polymerase, dNTPs, MgCl_2_ and loading dye, 1 μM of each primer, 5 μl of DNA template for the first reaction, and nuclease-free water up to the final volume. The second reaction was carried out with 1 μl of the first-round reaction product. Negative control was prepared with no template. DNA from an *in house* IDE8 tick cell line *E. canis* culture was used as positive control. Five microliters of each amplified product in the conventional PCR assays were subjected to horizontal electrophoresis in 1.5% agarose gel stained with Green Safe Premium (Nzytech) in 0.5x TEB run buffer (20 mM Tris, 20 mM boric acid, 0.5 mM EDTA, pH 7.2). Electrophoresis was performed at 100 V/400 W for 40 min. DNA Ladder VII molecular weight marker (NZYTech) was used to confirm the approximate size of the amplified products. The electrophoresis gel was imaged under ultraviolet light trans illuminator. Positive amplicons were purified using the NZYGelpure kit (NZYTech) and sent for Sanger sequencing at StabVida (Lisbon, Portugal). Obtained sequences were analyzed using DNASTAR software (www.dnastar.com) and Basic Local Alignment Search Tool (BLAST) used, for comparison with sequences deposited in the National Centre for Biotechnology Information (NCBI) nucleotide database (http://blast.ncbi.nlm.nih.gov/Blast).

### Proteome Analysis by Sequential Windowed Data Independent Acquisition of the Total High-Resolution Mass Spectra-Mass Spectrometry

Protein extracts (150 μg) were trypsin digested using the filter aided sample preparation (FASP) protocol (Wisniewski et al., 2009) with the FASP Protein Digestion Kit (Expedeon, TN, USA) following manufacturer recommendations. The resulting peptides were desalted onto OMIX Pipette tips C18 (Agilent Technologies, CA, USA), dried-down and stored at –20°C until mass spectrometry analysis. The desalted protein digests were resuspended in 2% acetonitrile, 5% acetic acid in water and analyzed by reverse phase liquid chromatography coupled to mass spectrometry (RP-LC-MS/MS) using an ekspertTM nanoLC 415 system on line with a 6600 TripleTOF^®^ mass spectrometer (AB SCIEX; Framingham, US) through Information-Dependent Acquisition (IDA) followed by Sequential Windowed data independent Acquisition of the Total High-resolution Mass Spectra (SWATH). Four micrograms of each protein digest of the four initial pools from each experimental group were joined together as a representative mixed sample of each of the eight experimental groups, which were used for the generation of the reference spectral ion library as part of SWATH-MS analysis.

The peptides were first concentrated in a 0.1 × 20 mm C18 RP precolumn (Thermo Scientific), and then separated using a 0.075 × 250 mm C18 RP column (New Objective, Woburn, MA, USA) operating at 0.3 µl/min. Elution of peptides were done in a 120-min gradient from 5 to 30% solvent B in solvent A followed by 15-min gradient from 30 to 60% solvent B in solvent A (Solvent A: 0,1% formic acid in water, solvent B: 0,1% formic acid in acetonitrile) and directly injected into the mass spectrometer for analysis. For IDA experiments, the mass spectrometer was set to scanning full spectra (350–1,400 m/z) using 250 ms accumulation time per spectrum, followed by up to 50 MS/MS scans (100–1,500 m/z). Candidate ions with a charge state between +2 and +5 and counts per second above a minimum threshold of 100 were isolated for fragmentation. One MS/MS spectra was collected for 100 ms, before adding those precursor ions to the exclusion list for 15s (mass spectrometer operated by Analyst^®^ TF 1.6, ABSciex^®^). Dynamic background subtraction was turned off. MSMS analyses were recorded in high sensitivity mode with rolling collision energy on and a collision energy spread of 5. For SWATH quantitative analysis, the four pools per group initially obtained were grouped in a single pool. Due to the low amount of protein obtained after extraction, three technical replicates per sample were performed in the case of non-infected samples and four technical replicates per sample for infected samples. These samples were subjected to the cyclic data independent acquisition (DIA) of mass spectra using the SWATH variable windows calculator (V 1.0, AB SCIEX) and the SWATH acquisition method editor (AB SCIEX), similar to established methods (Gillet et al., 2012). A set of 50 overlapping windows was constructed (containing 1 m/z for the window overlap), covering the precursor mass range of 400–1,250 m/z. For these experiments, a 50 ms survey scan (350–1,400 m/z) was acquired at the beginning of each cycle, and SWATH-MS/MS spectra were collected from 100 to 1500 m/z for 70 ms at high sensitivity mode, resulting in a cycle time of 3.6 s. Collision energy for each window was determined according to the calculation for a charge +2 ion-centered upon the window with a collision energy spread of 15.

### Library Generation/Protein Identification, Data Processing, and Relative Quantitation

A spectral library of all the detectable peptides in the samples was created and data processing was performed according to Villar et al. (2020), with the exception of spectra identification which was performed by searching against a compiled database containing all sequences from *Ixodidae* and *Ehrlichia* taxonomies and dog (*Canis lupus familiaris*) proteome (148,796, 19,528, and 25,491 Uniprot entries, respectively, in October, 2018) with the following parameters: iodoacetamide cysteine alkylation, trypsin digestion, identification focus on biological modification, and thorough ID as search effort. Positive identifications were considered when identified proteins reached a 1% of Global FDR. After SWATH processing, relative quantitation was performed in the Mar the MarkerView 1.3 software (Ab Sciex; https://sciex.com/products/software/markerview-software). Global normalization was performed according to the Total Area Sums (TAS) of all detected proteins in the samples. The Student’s t-test (p < 0.05) was used to perform two-sample comparisons between the averaged TASs of all the transitions derived from each protein across the replicate runs for each sample under comparison in order to identify proteins that were significantly differentially represented between groups. The MS raw proteomics data have been deposited at the PeptideAtlas repository (http://www.peptideatlas.org/) with the dataset identifier PASS01601.

### Functional Characterization and Protein Classification

The identified proteins in each dataset, were classified into the functional terms biological process (BP), molecular function (MF), and cellular component (CC) according to the Gene Ontology hierarchy (GO), using Blast2GO software (version 3.0.11, available at http://www.blast2go.org) (Conesa et al., 2005; Götz et al., 2008). Blast against Arthropoda (nr subset) [arthropoda, taxa: 6656] from 30.01.2017 was performed to achieve homology to the protein identification (UniprotID), mapping and annotation steps to assign functional terms at level 3. GO terms were also reviewed and manually annotated using the UniProt tools (http://www.uniprot.org) and the Protein Analysis Through Evolutionary Relationships (PANTHER) (http://www.pantherdb.org) classification system. The Microsoft Office 2016 Excel tool was used to construct the functional categorization charts based on the protein frequency in each GO category, for each experimental group.

## Results

### Molecular Confirmation of Non-infected and *Ehrlichia canis*-Infected Conditions

A nested PCR targeting a fragment of the *16S* rRNA gene confirmed the absence of *E. canis* DNA in all non-infected groups. The presence of *E. canis 16S* rDNA in the infected groups resulted in the validation of infection in three SG pools and three MG pools of *R. sanguineus* females (temperate lineage) as well as in four SG pools and two MG pools of *R. sanguineus* females (tropical lineage) (**Supplementary Figure 1**). The obtained sequences were trimmed, and a representative sequence was deposited in GenBank under the accession number MN719398. The sequences presented a query cover of 100%, an E value of 7^−161^, and showed 100% identity with sequences of *E. canis* (accession numbers MN396361, MN484597).

### Comprehensive Analysis of Non-Infected *Rhipicephalus sanguineus* s.l. Global Proteome

#### Sialome

The protein content of SG from non-infected *R. sanguineus* tropical lineage *versus* non-infected *R. sanguineus* temperate lineage females was analyzed. A total of 1,354 proteins were identified: while 73 (5.39%) corresponded to the vertebrate host, 1,281 (94.61%) were associated to *R. sanguineus*. No protein related with the pathogen was identified. All proteins of tick SG were annotated for each UniProtID using Blast2GO and UniProt-related databases. Sixty-five (5.08%) proteins were classified as “unknown” due to the absence of gene ontology and domain function annotation. Out of 1,281 proteins identified, 793 (61.90%) were differentially represented (p < 0.05) when the two different lineages were compared to each other: 431 (54.35%) over-represented and 362 (45.65%) under-represented. While the log_2_-normalized fold-change between proteins from SG of *R. sanguineus* tropical lineage *vs*. temperate lineage in a non-infected condition ranged from −8.33 to −0.12, for the under-represented proteins, a range of 0.09 to 6.41 was found for the over-represented proteins (**Supplementary Table 1**).

**Figure 2:**
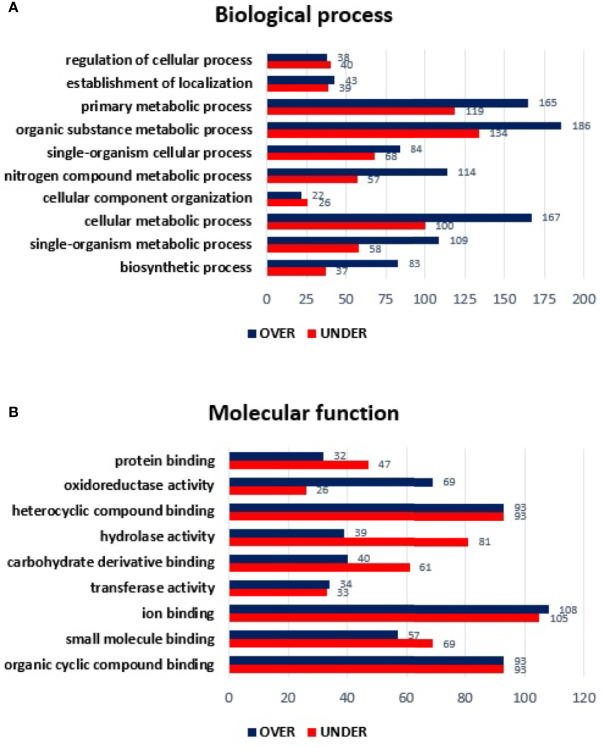
Gene ontology of differentially represented proteins from salivary glands of *R. sanguineus* tropical lineage *vs. R. sanguineus* temperate lineage in the non-infection condition. GO terms and its representation of biological process **(A)**, and molecular function **(B)** were obtained using level 3. Blue bars represent the number of over-represented proteins in the tropical lineage compared to the temperate lineage and red bars represented the number of under-represented proteins in the tropical lineage compared to the temperate lineage (can be interpreted as the number of proteins more represented in the temperate lineage compared to the tropical lineage), with statistical significance (*p* < 0.05).

Regarding the classification for BP, although ten GO terms were identified among the differentially represented proteins (**Figure 2A**), only the regulation of cellular process and cellular component organization processes showed to be more represented in the temperate lineage. The remaining categories showed higher number of over-represented proteins, with more pronounced differences in cellular metabolic process, followed by nitrogen compound metabolic process and organic substance metabolic process, which were found to be more represented in the tropical lineage.

Concerning the classification of MF, nine GO terms were identified in both lineages. Within the MF category, three GO terms (ion binding, transferase activity, and mainly oxidoreductase activity) showed a higher number of over-represented than under- represented proteins and four GO terms showed a higher number of under-represented proteins (proteins binding, carbohydrate derivative binding, and mainly hydrolase activity). Moreover, two GO terms (heterocyclic compounding binding and organic cyclic compounding binding) showed the same number of over and under-represented proteins (**Figure 2B**).

#### Mialome

**Figure 3:**
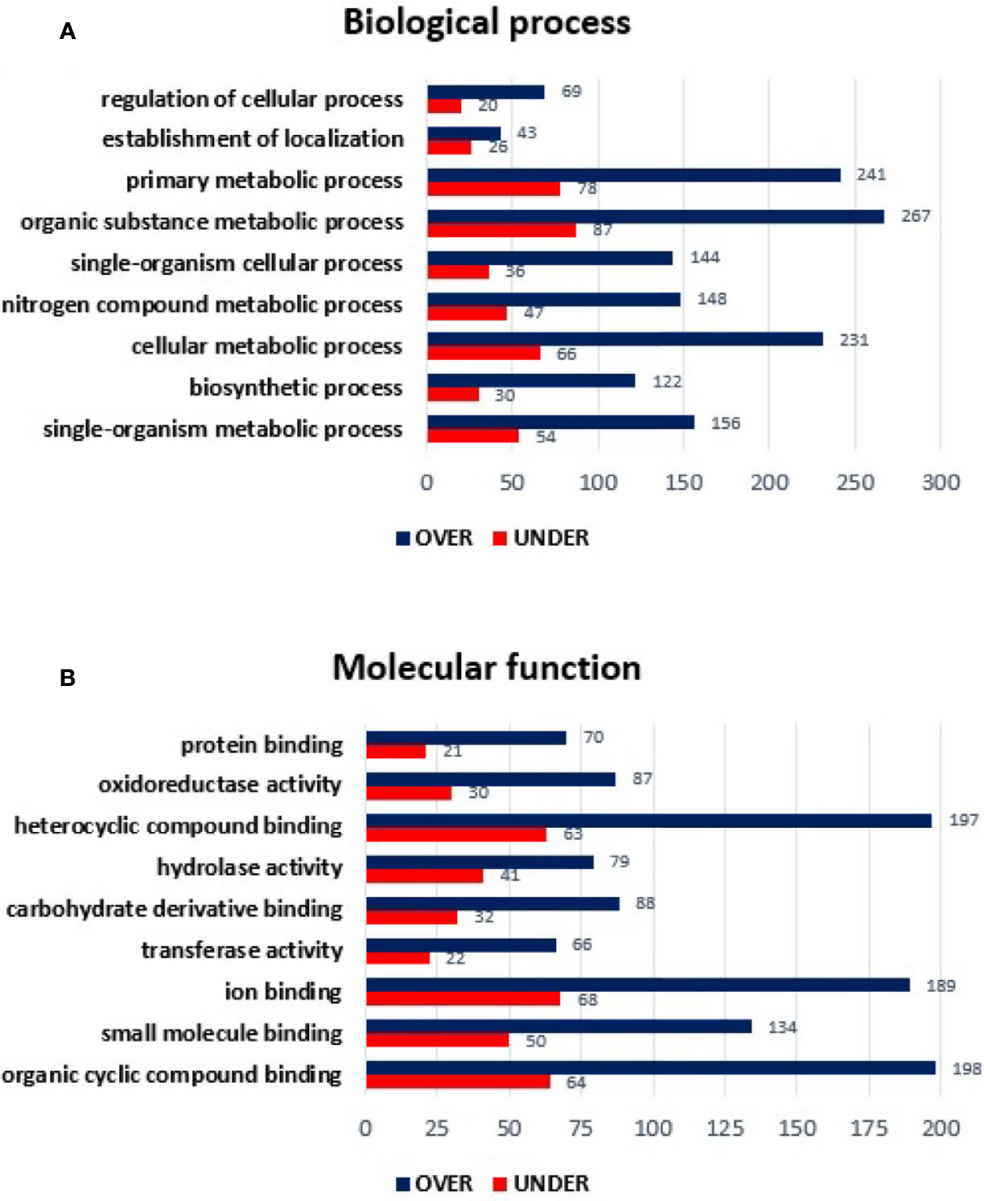
Gene ontology of differentially represented proteins from midgut of *R. sanguineus* tropical lineage *vs. R. sanguineus* temperate lineage in the non-infection condition. GO terms and its representation of biological process **(A)**, and molecular function **(B)** obtained using level 3. Blue bars represent the number of over-represented proteins in the tropical lineage compared to the temperate lineage and red bars represented the number of under-represented proteins in the tropical lineage compared to the temperate lineage (can be interpreted as the number of proteins more represented in the temperate lineage compared to the tropical lineage), with statistical significance (*p* < 0.05).

Using the same approach, MG from non-infected *R. sanguineus* tropical lineage *versus R. sanguineus* temperate lineage females were analyzed to infer about differences on protein content between the two groups. A total of 1,481 proteins were identified: while 152 (10.26%) corresponded to the vertebrate host, 1,329 (89.73%) were associated to tick proteins. No protein related with the pathogen was identified. After exclusion of host related proteins from the dataset, 73 (5.49%) tick MG proteins were classified as “unknown” due to the absence of GO and domain function annotation. Out of 1,329 proteins identified, 863 (64.93%) were differentially represented (p < 0.05), among 606 (70.22%) over-represented and 257 (29.77%) under-represented. While the log_2_-normalized fold-change ranged from −7.27 to −0.05 for the under-represented proteins, it ranged from 0.09 to 6.31 for the over-represented proteins (**Supplementary Table 2**). Classification of these differentially represented proteins according to BP resulted in the identification of nine BP, all showing a higher number of over-represented than under-represented proteins, with more pronounced differences in organic substance metabolic process, primary metabolic process, and cellular metabolic process (**Figure 3A** **)**. The same tendency was observed in the MF classification, since all categories showed a higher number of over-represented than under-represented proteins, with more pronounced differences in heterocyclic compounding binding, organic cyclic compounding binding, and ion binding (**Figure 3B** **)**.

### Analysis of *Ehrlichia canis*-Infected *Rhipicephalus sanguineus* s.l. Global Proteome

#### Sialome

**Figure 4:**
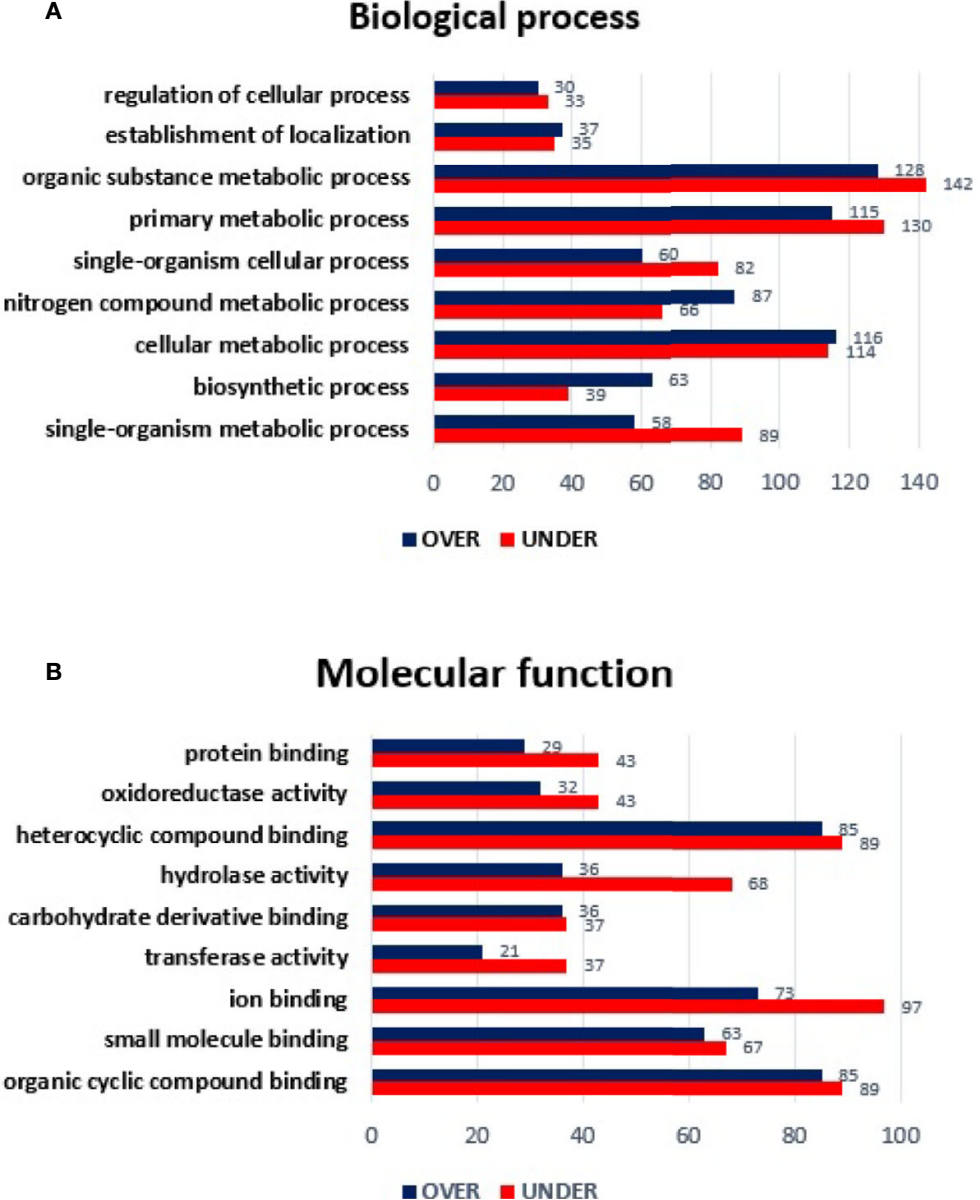
Gene ontology of differentially represented proteins from salivary glands of *R. sanguineus* tropical lineage *vs. R. sanguineus* temperate lineage in the infection condition. GO terms and its representation of biological process **(A)**, and molecular function **(B)** obtained using level 3. Blue bars represent the number of over-represented proteins in the tropical lineage compared to the temperate lineage and red bars represented the number of under-represented proteins in the tropical lineage compared to the temperate lineage (can be interpreted as the number of proteins more represented in the temperate lineage compared to the tropical lineage), with statistical significance (*p* < 0.05).

The SG proteome of *E. canis*-infected *R. sanguineus* tropical lineage *versus R. sanguineus* temperate lineage females was also analyzed to infer about differences on protein content in a condition of infection. A total of 908 proteins were identified: while 2 (0.22%) corresponded to the pathogen, 37 (4.07%) corresponded to the vertebrate host, and 869 (95.70%) to *R. sanguineus*. As previously mentioned, prior to further analysis, vertebrate host and pathogen proteins were removed from the dataset. Out of 869 proteins associated with tick SG, 65 (7.47%) proteins were classified as “unknown” due to the absence of GO and domain function annotation, whereas 643 (73.99%) were found differentially represented (p < 0.05), among 287 (44.63%) over-represented and 356 (55.36%) under-represented. The log_2_ normalized fold-change between SG proteins from *E. canis*-infected *R. sanguineus* tropical lineage *vs*. temperate lineage ranged from −4.81 to −0.09 for the under-represented proteins and from 0.07 to 9.01 for the over-represented proteins (**Supplementary Table 3**). Regarding BP, nine GO terms were identified: organic substance metabolic process, primary metabolic process, cellular metabolic process, nitrogen compound metabolic process, single organism metabolic process, single-organism cellular process, biosynthetic process, establishment of location, and regulation of cellular process. Out of these, four GO terms (cellular metabolic process, establishment of location, and mainly biosynthetic process and nitrogen compound metabolic process) showed a higher representation on the tropical lineage. On the other hand, regulation of cellular process, organic substance metabolic process, primary metabolic process, and single-organism cellular process, and single-organism metabolic process showed a higher number of representatives on the SG of *R. sanguineus* temperate lineage (**Figure 4A** **)**.

All MF GO terms identified herein showed a higher number of under-represented than over-represented proteins in the tropical lineage with more pronounced differences in hydrolase activity and ion binding (**Figure 4B** **)**.

#### Mialome

**Figure 5:**
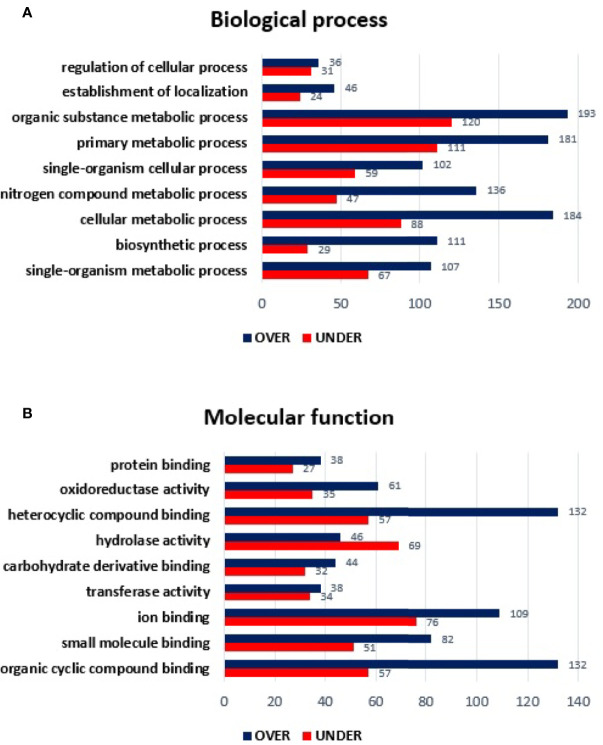
Gene ontology of differentially represented proteins from midgut of *R. sanguineus* tropical lineage *vs. R. sanguineus* temperate lineage in the infection condition. GO terms and its representation of biological process **(A)**, and molecular function **(B)** obtained using level 3. Blue bars represent the number of over-represented proteins in the tropical lineage compared to the temperate lineage and red bars represented the number of under-represented proteins in the tropical lineage compared to the temperate lineage (can be interpreted as the number of proteins more represented in the temperate lineage compared to the tropical lineage), with statistical significance (*p* < 0.05).

Regarding the MG from *E. canis*-infected *R. sanguineus* tropical lineage *versus R. sanguineus* temperate lineage females, a total of 1,078 proteins were identified: 91 (8.44%) corresponded to the vertebrate host and 987 (91.55%) to *R. sanguineus*. Due to the absence of GO and domain function annotation, 65 (6.58%) proteins were classified as “unknown.” Out of 987 proteins identified, 762 (77.20%) were differentially represented (p < 0.05), being 446 (58.53%) over-represented and 316 (41.46%) under-represented. The log_2_ normalized fold-change between MG proteins of both lineages in infected condition ranged from −5.45 to −0.08 for the under-represented proteins and from 0.07 to 7.08 for the over-represented proteins (**Supplementary Table 4**). GO analysis resulted in the identification of nine BP: the most abundant categories were organic substance metabolic process of (18.96%), primary metabolic process (17.63%), and cellular metabolic process (16.25%), followed by single organism metabolic process (10.78%), nitrogen compound metabolic process (10.45%), single-organism cellular process (9.88%), biosynthetic process (7.84%), establishment of location (4.08%), and regulation of cellular process (4.08%). All categories showed a higher number of over-represented than under-represented proteins, with more pronounced differences in cellular metabolic process, nitrogen compound metabolic process, and biosynthetic process (**Figure 5A** **)**. Regarding MF, only hydrolase activity showed a higher number of under-represented than over-represented proteins in the tropical lineage when compared to the temperate lineage. All other categories showed a higher number of over-represented than under-represented proteins, with more pronounced differences in organic cyclic compound binding and in heterocyclic compound binding (**Figure 5B** **)**.

### Proteins Present Only in the Infection Condition

#### Proteins Identified in Salivary Glands

**Figure 6:**
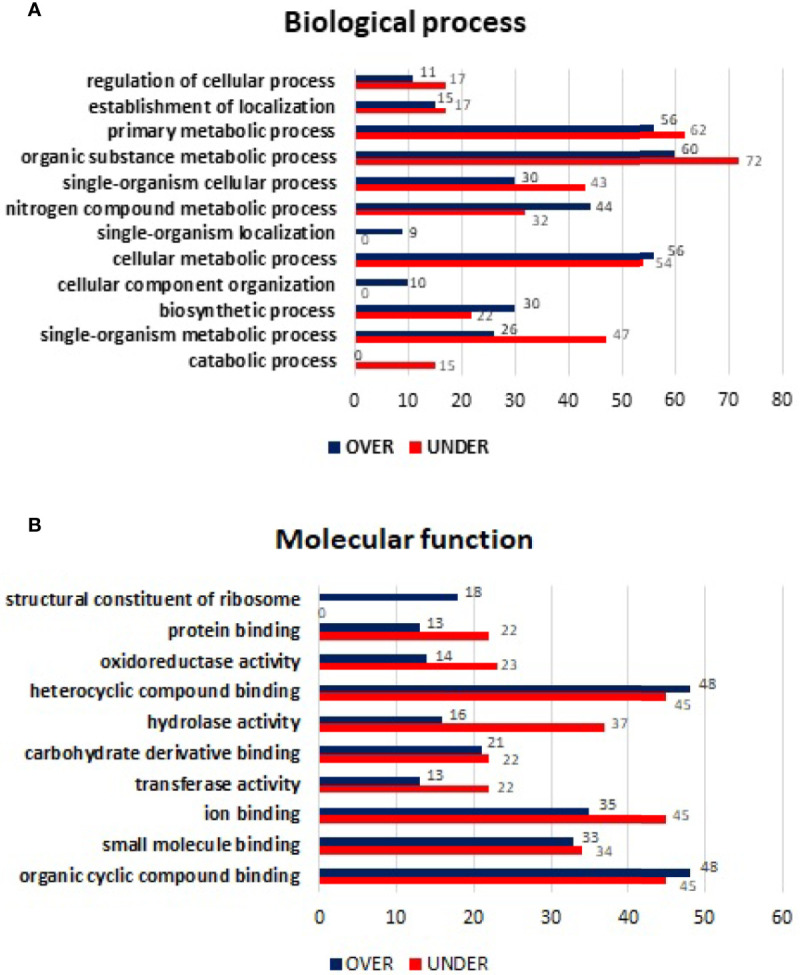
Gene ontology of differentially represented proteins from salivary glands of *R. sanguineus* tropical lineage *vs. R. sanguineus* temperate lineage identified only in the infection condition. GO terms and its representation of biological process **(A)**, and molecular function **(B)** obtained using level 3. Blue bars represent the number of over-represented proteins in the tropical lineage compared to the temperate lineage and red bars represented the number of under-represented proteins in the tropical lineage compared to the temperate lineage (can be interpreted as the number of proteins more represented in the temperate lineage compared to the tropical lineage), with statistical significance (*p* < 0.05).

From the 643 differentially represented proteins of *R. sanguineus* tropical lineage *vs*. temperate lineage SG in infected condition, 335 proteins were identified only in infected condition of which, 142 were over-represented and 193 under-represented. The log_2_-normalized fold-change between proteins from SG of the *R. sanguineus* tropical lineage *vs*. temperate lineage identified only in *E. canis*-infected condition ranged from −4.69 to −0.09 for the under-represented proteins and from 0.10 to 9.01 for the over represented proteins (**Supplementary Table 5**). Annotation of proteins found only in tick SG from the infected condition resulted in the identification of twelve BP and ten MF terms (**Figure 6** **).** Particularly, regarding BP (**Figure 6A**), the nitrogen compound metabolic process and biosynthetic process showed a higher number of over-represented than over-represented proteins. On the other hand, regulation of cellular process, establishment of location process, primary metabolic process, organic substance metabolic process, and single organism metabolic process showed a higher number of under-represented than over-represented proteins. Cellular component organization and single organism organization were identified only with over-represented proteins, while the catabolic process category was identified only with under-represented proteins. Regarding MF (**Figure 6B** **)**, only heterocyclic compound binding and organic cyclic compound binding showed a higher number of over-represented than under-represented proteins, while protein binding, oxidoreductase activity, hydrolase activity, carbohydrate derivative binding, transferase activity, ion binding, and small molecule binding showed a higher number of under-represented than over-represented proteins, with more pronounced difference in hydrolase activity. The GO term structural constituent of ribosome was identified only with over-represented proteins.

Proteins with log_2_-normalized fold-change ≤−4 or ≥4 were selected to perform a comprehensive analysis. Among the ten proteins that attended this criterion, seven presented log_2_-normalized fold-change ≥4, namely, two putative cement proteins (UniProt ID: C9W1K5, and C9W1A6), a putative cuticle protein (UniProt ID: B7P5Q1), a putative transglutaminase/protease-like protein (UniProt ID: A0A131XLJ3), a TIL domain containing protein (UniProt ID: A0A224YL44), a cytochrome b-c1 complex subunit 6 (UniProt ID: B7PEW1), and a secreted protein (UniProt ID: C9W1N5). On the other hand, only three proteins showed log_2_-normalized fold-change ≤−4, namely, putative secreted salivary gland peptide (UniProt ID: A0A131XAM3), putative glutathione s-transferase (UniProt ID: A0A023G883), and cystatin (UniProt ID: A0A131Z4A2). Complete information about UniProt ID, description, and log_2_-normalized fold-change is presented in **Table 1**.

**Table 1:** Proteins identified in salivary glands of *R. sanguineus* from the tropical lineage *vs. R. sanguineus* from temperate lineage identified only in the infection condition, with log_2_-normalized fold-change ≤−4 or ≥4.

UniProt ID	Description	Organism	Fold change (Log-_2_)
C9W1K5	Putative cement protein	*R. sanguineus*	9.006074
C9W1A6	Putative cement protein	*R. sanguineus*	8.729295
B7P5Q1	Cuticle protein	*I. scapularis*	6.350449
A0A131XLJ3	Putative transglutaminase/protease-like protein	*H. excavatum*	5.09856
A0A224YL44	TIL domain containing protein	*R. zambeziensis*	4.873546
B7PEW1	Cytochrome b-c1 complex subunit 6	*I. scapularis*	4.151654
C9W1N5	Secreted protein	*R. sanguineus*	4.136042
A0A131XAM3	Putative secreted salivary gland peptide	*H. excavatum*	-4.07746
A0A023G883	Putative glutathione s-transferase	*A. triste*	-4.15374
A0A131Z4A2	Cystatin	*R. appendiculatus*	-4.69022

#### Proteins Identified in Midgut

**Figure 7:**
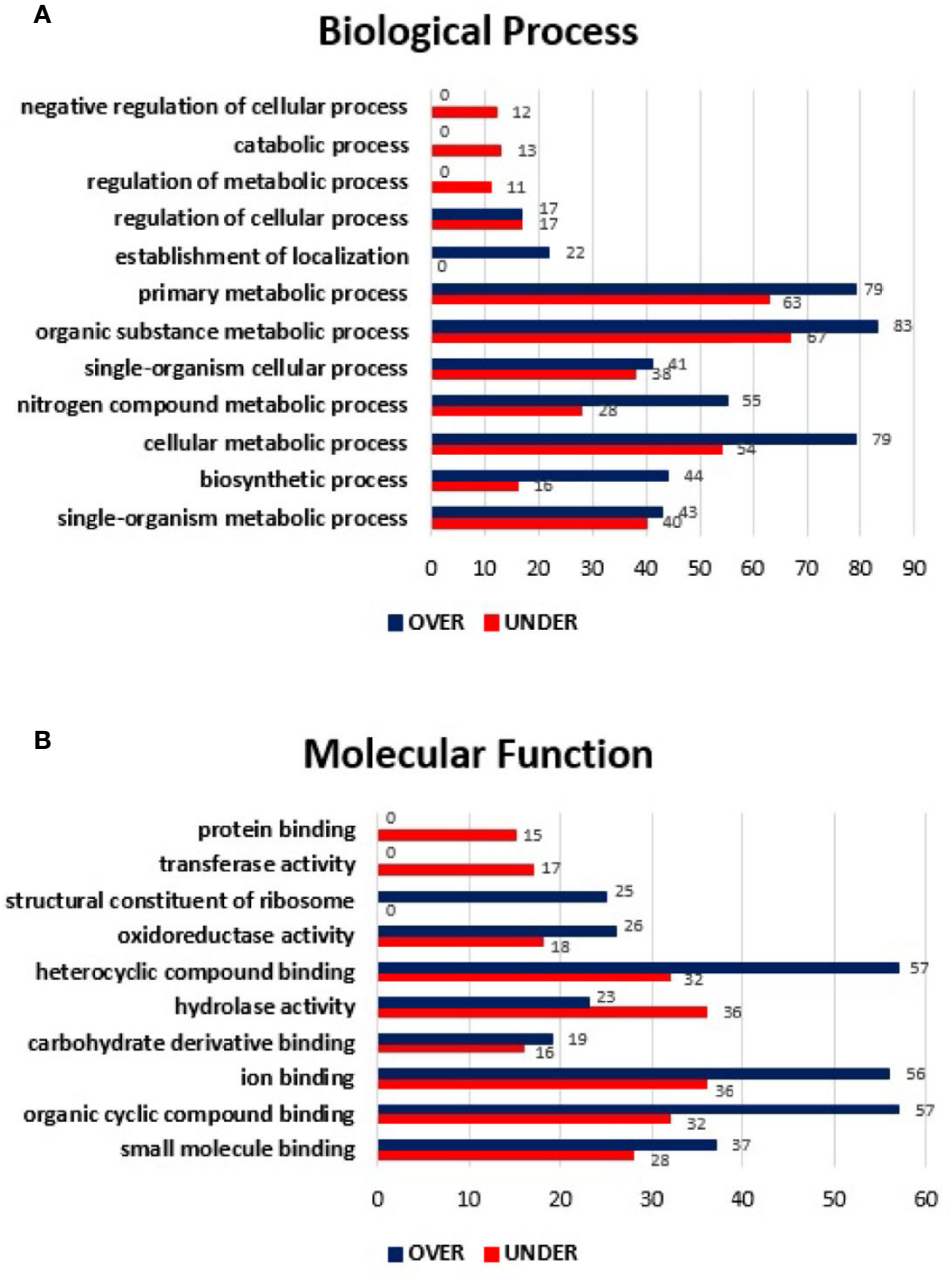
Gene ontology of differentially represented proteins from midgut of *R. sanguineus* tropical lineage *vs. R. sanguineus* temperate lineage identified only in the infection condition. GO terms and its representation of biological process **(A)**, and molecular function **(B)** obtained using level 3. Blue bars represent the number of over-represented proteins in the tropical lineage compared to the temperate lineage and red bars represented the number of under-represented proteins in the tropical lineage compared to the temperate lineage (can be interpreted as the number of proteins more represented in the temperate lineage compared to the tropical lineage), with statistical significance (*p* < 0.05).

Regarding the 762 differentially represented MG proteins, 374 proteins were found only in the *E. canis*-infected condition; of which, 206 were over-represented and 168 under-represented. The log_2_-normalized fold-change between MG proteins of the *R. sanguineus* tropical lineage *vs*. temperate lineage identified only in-infected condition ranged from −5.35 to −0.14 for the under-represented proteins and from 0.10 to 6.78 for the over-represented proteins (**Supplementary Table 6**). Regarding BP, primary metabolic process, organic substance metabolic process, single-organism metabolic process, nitrogen compound metabolic process, cellular metabolic process, biosynthetic process, and single-organism cellular process showed a higher number of over-represented than under-represented proteins, with more pronounced difference in biosynthetic process, cellular metabolic process, and nitrogen compound metabolic process. While the GO term establishment of localization was identified only with over-represented proteins, the terms negative regulation of catabolic process, catabolic process, and regulation of metabolic process were identified only with under-represented proteins (**Figure 7A** **).**

Concerning the MF, while structural constituent of ribosome was a GO term only identified with over-represented proteins, the GO terms protein binding and transferase activity were identified only with under-represented proteins (**Figure 7B** **).**

Similarly, midgut proteins with log_2_-normalized fold-change ≤−4 or ≥4 were selected to perform a comprehensive analysis. Among the nine proteins that met this criterion, four presented log_2_-normalized fold-change ≥4, namely a metastriate one of each protein family (UniProt ID: A0A131YMZ), putative cement protein (UniProt ID: C9W1K5), phosphoinositide phospholipase C (UniProt ID: L7MKF4), and a putative glycine-rich cell wall structural protein (UniProt ID: A0A023FM06). On the other hand, five proteins showed log_2_-normalized fold-change ≤−4, namely, an RNA-binding protein LARK (UniProt ID: A0A224Z0H9), a microsomal epoxide hydrolase (UniProt ID: A0A2245Z4E8), a putative secreted salivary gland peptide (UniProt ID: A0A131XAM3), a 2,3-biphosphoglycerate-independent phosphoglycerate mutase (UniProt ID: A0A224YRN6), and cystatin (UniProt ID: A0A1E1X068). Complete information about UniProt ID, description, and log2-normalized fold-change is presented in **Table 2**.

**Table 2:** Proteins identified in the midgut of *R. sanguineus* from the tropical lineage *vs. R. sanguineus* female from temperate lineage identified only in the infection condition, with log_2_-normalized fold-change ≤−4 or ≥4.

UniProt ID	Description	Organism	Fold change (Log-_2_)
AOA131YMZ1	Metastriate one of each protein family	*R. appendiculatus*	6.783897
C9W1K5	Putative cement protein	*R. sanguineus*	4.95425
L7MKF4	Phosphoinositide phospholipase C	*R. pulchellus*	4.137119
A0A023FM06	Putative glycine-rich cell wall structural protein	*A. cajennense*	4.06581
A0A224Z0H9	RNA-binding protein LARK	*R. zambeziensis*	−4.04922
A0A224Z4E8	Microsomal epoxide hydrolase	*R. zambeziensis*	−4.44118
A0A131XAM3	Putative secreted salivary gland peptide	*H. excavatum*	−4.9514
A0A224YRN6	2,3-bisphosphoglycerate-independent phosphoglycerate mutase	*R. zambeziensis*	−5.01535
A0A1E1X068	Cystatin	*A. aureolatum*	−5.34675

## Discussion

### Comparative Proteomics Supports the Separation of *Rhipicephalus sanguineus* Lineages Into Distinct Taxonomic Units

A different protein representation pattern was observed in unfed pathogen-free *R. sanguineus* females from the tropical *versus* the temperate lineage. These data added to those already known about the differences between these two lineages observed on morphology, genetics, biology, and behavior, supporting the hypothesis that these lineages represent distinct taxonomic units *i.e.* species (Szabó et al., 2005; Sanches et al., 2016; Labruna et al., 2017; Nava et al., 2018). A higher number of over-represented proteins than under-represented were observed in both SG and MG, suggesting that in the tropical lineage there is a greater translational investment in comparison with the temperate lineage. Most of these proteins were grouped in GO terms related with metabolic processes, which suggests that again the tropical lineage is more metabolically active than the temperate lineage. This may be a consequence of different evolutionary survival strategies, which synchronize the life cycle with favorable abiotic conditions, and availability of hosts. In fact, the tropical lineage completes two to four generations per year, whereas the temperate completes only one generation in the same period of time, under natural conditions (Labruna et al., 2017). Unfed adults of *R. sanguineus* from the temperate lineage that molted from nymphs under summer conditions remain dormant for some time after post molting while the tropical lineage takes less time to become aggressive (Labruna et al., 2017). It is therefore expected that the tropical lineage consumes the gut reserve content more quickly and use this energy to search for a new host in less time after molting than the temperate lineage. Accordingly, in *R. sanguineus* tropical lineage, the putative F0F1-type synthase gamma subunit (UniProt ID: L7M679) was the most over-represented protein in both tissues, with a log_2_-normalized fold-change of 6.31 and 6.42, respectively. This protein is associated with the energy metabolism, catalyzing the synthesis of ATP and recently, its importance on the physiology of *Haemaphysalis longicornis* female ticks was demonstrated, whereas F0F1 ATP synthase knocked down by RNAi, resulted in morphological changes of the acini of SG, a reduction of weight in engorged females and impairment of oviposition (Usukura et al., 2012; Wang et al., 2019). This protein represents an interesting target to be studied in *R. sanguineus* due its potential as vaccine candidate.

A noteworthy group of proteins, considered one of the most abundant salivary components in ticks, are the lipocalins (Sangamnatdej et al., 2002). These represent a wide group of small extracellular molecules associated with the process of modulation of host immune response, such as the inflammatory response, playing important role during tick feeding (Beaufays et al., 2008; Wang et al., 2016; Contreras et al., 2017). In the present study, most of the identified lipocalins were found to be under-represented in *R. sanguineus* tropical lineage. In SG, the most under-represented proteins of this class were a putative group 2 salivary lipocalin (UniProt ID: L7MBT5), followed by a putative salivary lipocalin (UniProt ID: L7LR20), and a lipocalin (UniProt ID: A0A131Z2K7), with a log_2_-normalized fold-change of -8.33, -5.90, and -5.38, respectively. In contrast, an evasin (UniProt ID: A0A131YUQ8) was found over-represented in the SG of *R. sanguineus* tropical lineage, with a log2-normalized fold-change of 5.26. These results suggest that the two *R. sanguineus* lineages have different adaptive tick-host interactions since the temperate lineage invests more in lipocalin production than the tropical lineage. This may be related to the ability of this lineage to ingest greater volumes of blood, often with a longer period of engorgement (Sanches et al., 2016). Moreover, lipocalins may have a dual role in tick-pathogens interaction: may facilitate pathogens transmission reducing host inflammatory response and, on the contrary, control tick infection by depleting strategic compounds for pathogens (Valdés et al., 2016). The different representation of lipocalins also may contribute to both lineages to react differently to infection by distinct pathogens.

### Pressure of Infection: Comparison of Proteomic Profiles Suggests That *Rhipicephalus sanguineus* Tropical Lineage Is More Tolerant to *Ehrlichia canis* Infection

The proteome of both *R. sanguineus* lineages during *E. canis* infection was compared to determine potential differences in the tick response to infection. The SG and MG obtained from 30-days-old *R. sanguineus* females of the tropical and the temperate lineages were screened for infection by nested PCR (Murphy et al., 1998). Detection on both tissues indicates that the pathogen is able to cross the MG barrier in both *R. sanguineus* lineages and reach the SG. However, since vector competence is determined by a combination of factors and not only the susceptibility to pathogen infection, the presence of *E. canis* in SG of the temperate lineage does not necessarily means that this lineage can be an *E. canis* vector. This suggestion was studied by Moraes-Filho et al. (2015), who demonstrated that this lineage was not able to transmit *E. canis* to dogs. Assuming that the live bacteria can reach SG but transmission does not occur to the vertebrate host, in the temperate lineage ticks, suggests the existence of a different cellular approach towards the pathogen. Future studies focusing SG components, such as antimicrobial proteins with the ability to kill Gram-positive and Gram-negative bacteria, are needed to deepen our understanding on the mechanisms involved in the transmission of *E. canis*.

A comparative proteomic analysis performed for the two lineages in relation to infection, showed that proteins related to hydrolase activity are more under-represented in the *R. sanguineus* tropical lineage in contrast to what was observed in the non-infection condition. Proteins with hydrolase activity are known to play an important role in metabolism of xenobiotic, including pathogens and pesticides, contributing to the organism detoxification (Hodgson and Rose, 2008). With respect to SG, proteins with MF related to oxidoreductase and transferase activities are more represented in the temperate lineage, suggesting that this lineage has a stronger response to infection. Tick cells increase the production of proteins related with oxidoreductase activity such as oxidative oxygen species (ROS) to limit *Anaplasma phagocytophilum* infection, whilst some proteins, associated with the transferase activity, play an important role in the detoxification of xenobiotic compounds due to oxidative stress in response to infection (Alberdi et al., 2019). On the other hand, under-representation of such proteins in the tropical lineage may indicate that the pathogen attempts to subvert the cellular response at SG level ensuring its survival. In addition, results demonstrate that in infection condition, *R. sanguineus* metabolism is directed differently in each lineage. According to Cabezas-Cruz et al. (2019), the response of tick cells to the pathogens is associated with tolerance to infection and, the modulation of tick metabolism by tick-borne pathogens, is a result of coevolution and adaptation, indicating that both *R. sanguineus* lineages had a different co-evolution with *E. canis.* There are many proteins involved in metabolic processes, however, the approach used in this study (comparison between the two different lineages) do not allow to predict how the pathogen exploit these molecules. For this purpose, a proteomic analysis should be performed comparing the same lineage in non-infected and infected conditions.

### Proteins Present Only in the Infected Condition

#### The Importance of the Cement Cone on Pathogen Transmission

To get a better understanding of *R. sanguineus* vector competence to *E. canis*, the proteins only present in the infection condition were analyzed in both SG and MG. In SG, four of the most represented proteins play important roles in the cement cone structure and function: two putative cement proteins (UniProt ID: C9W1K5 and C9W1A6), a cuticle protein (UniProt ID: B7P5Q1) and a putative transglutaminase/protease-like protein (UniProt ID: A0A131XLJ3). The over-representation of these proteins indicates that a better cement structure may favor the pathogen transmission. The cement cone is an adhesive proteinaceous matrix secreted by the majority of hard ticks around the mouthparts, to assist in the attachment, avoiding being groomed off the host, as well as to protect the mouthparts from the host immune responses (Bullard et al., 2016; Suppan et al., 2018). The putative cement protein (UniProt ID: C9W1K5) was also identified in the MG. The presence of this protein in both organs suggests that it may be recycled from SG to MG and back to SG. Previous studies showed that genes encoding putative cement proteins in *R. bursa* were found to be up regulated in response to *Babesia ovis* infection, in accordance to other studies (Antunes et al., 2018), and that the silencing of a cement protein of *R. bursa* reduced the tick attachment, feeding process, and consequently, the female body weight. Therefore, the two proteins herein identified (UniProt ID: C9W1K5 and C9W1A6) represent a potential target for the development of a vaccine affecting both the vector and pathogen. The transglutaminase/protease-like protein (UniProt ID: A0A131XLJ3) may contribute to harden the cement cone acting as a crosslinking enzyme at the glutamine residues (Bullard et al., 2016; Bullard et al., 2019). The gene coding to transglutaminase was one of the most induced in *Dermacentor variabilis* in response to different bacteria (Jaworski et al., 2010). These class of proteins have been only poorly studied in ticks but recently have emerged as key molecules in insects’ vector of phytovirus (Deshoux et al., 2018). Studies indicated that insect cuticle proteins might facilitate virus entry at the gut barrier, could bind virus particles in the hemolymph and hence assist viral movement towards the SG (Cilia et al., 2011). Future research should be conducted to fill this knowledge gap on the role of cuticle proteins on tick-pathogen interactions.

In the MG, glycine-rich proteins (GRPs) were found has an infection exclusive protein (UniProt ID: A0A023FM06). Belonging to a superfamily of proteins characterized by the presence of glycines in repeated motifs or isolated along the primary amino acid sequence, GRPs have been identified in a wide variety of organisms, involved in diverse processes such cell wall structure, cuticle construction, cell elongation, signal transduction, defense and response to stress, RNA binding, and antimicrobial activity (Zhong et al., 2006; Zhang et al., 2008; Leal et al., 2018). In ticks, GRPs have been identified predominantly in SG, but also in other organs like synganglia and MG (Anderson et al., 2008; Leal et al., 2018). In SG, GRPs are associated to the cement cone formation that affords tick attachment to the host during initial feeding phase however, in the MG, their role remains unknown (Leal et al., 2018).

#### Energy and Protease Inhibitors Toward Pathogen Transmission

TIL-type protease inhibitors have been reported to inhibit proteases such as trypsin, cathepsin, elastase, and chymotrypsin, thus playing an important role in ticks physiological processes, improving parasitism mechanisms, and facilitating blood feeding by interfering with defense-related host peptidases (Shakeel and Zafar, 2020). Although information about this class of proteins, in insects and ticks, is limited, members of the TIL domain containing protein have been identified in blood-feeding insects and tick sialomes (Mans, 2011; Tirloni et al., 2014; Maruyama et al., 2017). A secreted cysteine-rich protein containing the TIL domain was upregulated in fed *Amblyomma sculptum* in comparison with unfed ticks (Esteves et al., 2017). Similarly, TIL domain-containing proteins were also upregulated in fully engorged *R. (B.) microplus* females, in comparison with partially engorged females (Tirloni et al., 2014). Moreover, the BmSI-17, a trypsin inhibitor-like cysteine-rich domain presented strong inhibitory activity to elastase, which participates in the cattle inflammatory response in consequence of tick bite (Sasaki et al., 2008). In this study, the trypsin inhibitor-like containing protein (UniProt ID: A0A224YL44) was found during infection only in SG, suggesting that *E. canis* manipulates *R. sanguineus* machinery in order to improve the feeding process to favor pathogen transmission. Additionally, this class of proteins may also have antimicrobial function, regulating the quantity and specificity of tick pathogens acquired and transmitted (Tirloni et al., 2014). In *R. (B.) microplus*, a protein of this group, ixodidin, was purified from the hemocytes and classified as an antimicrobial peptide, showing to affect *Micrococcus luteus* and *Escherichia coli* growth and inhibitory activity (Fogaça et al., 2006). The cytochrome *bc1* complexes (cytochrome *bc1* complex subunit 6, UniProt ID: B7PEW1) are conserved intrinsic membrane proteins that catalyze the oxidation of ubihydroquinone and the reduction of cytochrome c in mitochondrial respiratory chains, responsible for energy production in form of ATP (Fisher and Meunier, 2008). Previous studies have showed that pathogen infection modulates ATP production pathways meaning that targeting proteins considered relevant may be a useful way of controlling vectors and pathogens (Fisher and Meunier, 2008; Flores-Ramirez et al., 2019; Couto et al., 2020).

#### Detoxification and Defensive Response Modulation

The putative glutathione S-transferase (GST) (UniProt ID: A0A023G883) and the cystatin (UniProt ID: A0A131Z4A2) were also identified in SG as being exclusive of the response to infection.Our findings suggest that *E. canis* infection modulates the production of GST. The ubiquitinous GST family of proteins are involved in a wide range of biological and physiological processes, such as intracellular transport, biosynthesis of hormones, protection against oxidative stress, and regulation of apoptosis (Enayati et al., 2005). The effect of GSTs in detoxification of chemical components of acaricides were shown in *R. sanguineus*, *H. longicornis* and *R. microplus* (Duscher et al., 2014; Nandi et al., 2015; Hernandez et al., 2018). GST has been proposed as anti-tick vaccine due to encouraging results in reducing the number, weight and fertility of ticks post feeding on immunized hosts (Oldiges et al., 2016; Sabadin et al., 2017; Ndawula et al., 2019; Huercha et al., 2020).

Microsomal epoxide hydrolase (MEH) is an endoplasmic reticulum enzyme expressed in most tissues of a broad range of species (Beetham et al., 1995; Coller et al., 2001) and was herein found to be only expressed in response to infection in *R. sanguineus* MG (UniProt ID: A0A224Z4E8). This protein catalyzes the hydrolysis of epoxides to trans-dihydrodiols. Notably, the enzyme is able to either detoxify a wide variety of xenobiotics and genotoxins or promote the bio activation of substrates. In insects, most of the studies are focused on its involvement in the metabolism of a juvenile hormone, which regulates maturation, reproduction, behavior, diapause, and other biological aspects (Bede et al., 2001). In ticks, there are still no studies related to this class of proteins however, having in mind that orthologues encode to proteins showing similar functions, their role in the tick-pathogen interactions may be suggested.

Cystatin identified here (UniProt ID: A0A131Z4A2) is part of a large superfamily of proteins that act as natural inhibitor of papain-like cysteine and legumains found in a wide range of organisms including ticks (Schwarz et al., 2012; Wang et al., 2015). They are involved in various physiological processes, such as protein catabolism, regulation of hormone processing, inflammatory process, immune response, phagocytosis, and resistance to pathogen infections (Schwarz et al., 2012). In accordance to the high number of functions some cystatins can be found to be upregulated upon the infection and others downregulated (Maruyama et al., 2017). It was previously demonstrated that the expression of an HLcyst-2, a type 2 cystatin identified in *Haemaphysalis longicornis* increased when ticks were injected with *Babesia gibsoni*, and *in vitro* cultivation of *B. gibsoni* in the presence of Hlcyst-2 significantly inhibited pathogen growth, suggesting a role in tick immunity (Zhou et al., 2006). On the contrary, infection of *R. (B.) microplus* with *E. coli* promote the downregulation of Bmcystatin3 expression, but increased the efficacy of pathogen clearance, suggesting that this protein may also have a role as negative regulator of tick immune response (Lu et al., 2014). Thus, more studies should be conducted to assess the potential impact of the identified cystatin in both *R. sanguineus* lineages vector competence to *E. canis.*

#### Modulation of Cellular Response and Pathogen Entry/Reproduction

The protein phosphoinositide phospholipase C (UniProt ID: L7MKF4) was found in *R. sanguineus* MG but only during *E. canis* infection. The phosphoinositide-specific phospholipase C (PLC) isozymes have been identified in a broad range of organisms, including bacteria, yeast, plants, animals, and viruses (Rhee, 2001). They play important roles in cellular responses to a variety of extracellular signals, catalyzing the rapid hydrolysis of the phosphatidylinositol 4,5-bisphosphate, a minor membrane phospholipid, generating two intracellular messengers, diacylglycerol and inositol 1,4,5-trisphosphate, which activate the protein kinase C, and induce the release of calcium ions from intracellular stores (Rhee, 2001). In ticks, this class of proteins have been poorly studied, but may play an important role in tick-pathogen interactions. *E. chafeensis*, *E. canis, and A. phagocytophilum* proteins induce transglutamination, tyrosine phosphorylation, and PLC activation with consequent inositol 1,4,5-trisphosphate production, and increase in intracellular calcium, required for entry and reproduction of the pathogen in host cells (Rhee, 2001; Rikihisa, 2006).

## Conclusion

This comparative study highlights differences in proteomic profiles of SG and MG from the tropical and temperate lineages of *R. sanguineus* lineages, mainly in those proteins associated with metabolic processes. These findings in association with previously described genetic, biological, and behavioral traits, support the hypothesis of the existence of two distinct *R. sanguineus* species. Some proteins (e.g. those related to the cement cone and ATP metabolism/feeding process) found only during the *E. canis* infection could play an important role in favoring the *E. canis* infection in *R. sanguineus* tropical lineage. In contrast, proteins related with detoxification and defensive response modulation, which showed to be more represented in the temperate lineage of *R. sanguineus*, might contribute to reduce the vector competence of this lineage to *E. canis.* This newly acquired data shed some light on the understanding of molecular interactions between both *R. sanguineus* lineages and *E. canis*, and open avenues to further experiments to explore these proteins as potential targets to mitigate the *E. canis* infection in the *R. sanguineus* tropical lineage.

## Data Availability Statement

The datasets presented in this study can be found in online repositories. The names of the repository/repositories and accession number(s) can be found in the article/**Supplementary Material**.

## Ethics Statement

The animal study was reviewed and approved by Ethics Committee on Animal Experiments (CEUA) of the School of Agricultural and Veterinary Sciences of Universidade Estadual Paulista (UNESP), Jaboticabal, SP, Brazil, process number 000800/18.

## Author Contributions

GS, SA, JF, MV, and AD designed the study. GS and DB-B maintained the tick colony and produced the ticks used in the experiment. GB, RM, and MA conducted the experimental infection of the dogs with *E. canis*. MA and RM tested the dogs to ensure the absence of pathogens and performed the qPCRs to follow the course of the *E. canis* infection. GS dissected the salivary glands and midguts of ticks. GS, JF, and IM conducted the DNA and protein extractions and the validation of the infection in ticks by PCR. GS, LM-H, and MV performed the proteomics assay. GS, JC, MV, AD, and JF conducted the proteomic data analysis. GS, AD, and SA wrote the article. All authors contributed to the article and approved the submitted version.

## Funding

This research was supported by the project PTDC/CVT-WEL/1807/2014, subsidized by the Fundação para a Ciência e Tecnologia (FCT). The authors would like to acknowledge FCT for funds to GHTM – UID/Multi/04413/2013, Fundação de Amparo a Pesquisa do Estado de São Paulo - FAPESP (2018/06651-7), Conselho Nacional de Desenvolvimento Científico e Tecnológico –CNPq (421980/2016-8) and Coordenação de Aperfeiçoamento de Pessoal de Nível Superior – CAPES (88887.464801/2019-00). MA is a fellowship of CNPq (Productivity Grant - Process #302420/2017-7). JF and JC are fellowships of FCT (Grants SFRH/BD/122894/2016 and SFRH/BD/121946/2016).

## Conflict of Interest

The authors declare that the research was conducted in the absence of any commercial or financial relationships that could be construed as a potential conflict of interest.

## Supplementary Material

Supplementary Figure 1 Agarose 1.5% electrophoresis gel showing amplicons resulting from the second reaction of the nested PCR targeting a 396 bp fragment of *E. canis 16S rRNA* gene in salivary glands and midgut from the tropical (1–4) and temperate (5–8) lineages of 30 days post molting *R. sanguineus* females (nymphs were fed on an infected dog). M, 100-bp molecular weight marker lane; SG, salivary glands pool; MG, midgut pool; C+, positive control lane; C−, negative control lane.

Supplementary Table 1 Analysis of the sialome of non-infected *R. sanguineus* tropical *vs.* temperate lineage.

Supplementary Table 2 Analysis of the mialome of non-infected *R. sanguineus* tropical *vs.* temperate lineage.

Supplementary Table 3 Analysis of the sialome of *R. sanguineus* tropical vs. temperate lineage in *E. canis* infected condition.

Supplementary Table 4 Analysis of the mialome of *R. sanguineus* tropical vs. temperate lineage in *E. canis* infected condition.

Supplementary Table 5 Proteins identified only in *E. canis* infected SG of the *R. sanguineus* tropical lineage *vs*. temperate lineage.

Supplementary Table 6 Proteins identified only in *E. canis* infected MG of the *R. sanguineus* tropical lineage *vs*. temperate lineage.
