# Supplementary figures and images for: Comparative Proteomic Analysis of Rhipicephalus sanguineus sensu lato (Acari: Ixodidae) Tropical and Temperate Lineages: Uncovering Differences During Ehrlichia canis Infection
Source: Front Cell Infect Microbiol. 2021 Jan 29;10:611113. doi: 10.3389/fcimb.2020.611113 (PMC7879575; doi:10.3389/fcimb.2020.611113)

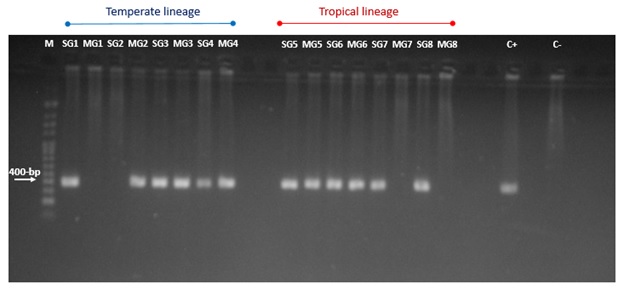

Supplement: Supplementary Figure 1 — Agarose 1.5% electrophoresis gel showing amplicons resulting from the second reaction of the nested PCR targeting a 396 bp fragment of E. canis 16S rRNA gene in salivary glands and midgut from the tropical (1–4) and temperate (5–8) lineages of 30 days post molting R. sanguineus females (nymphs were fed on an infected dog). M, 100-bp molecular weight marker lane; SG, salivary glands pool; MG, midgut pool; C+, positive control lane; C−, negative control lane. [file Image_1.jpeg]
